# Computational Screening of Djiboutian Medicinal Plants Reveals Potential Dual Inhibitors Against *Plasmodium falciparum* and *Plasmodium vivax*

**DOI:** 10.3390/cimb48070701

**Published:** 2026-07-10

**Authors:** Fatouma Mohamed Abdoul-Latif, Lamiae El Bouamri, Badr Sellami, Amal Bouribab, Fatimazahra Guerguer, Houda Mohamed, Abdirahman Elmi, Yahya Ali Ismae, Ricardo Gil-Ortiz, Samir Chtita

**Affiliations:** 1 Medicinal Research Institute, Center for Research and Study of Djibouti, Djibouti City 77101, Djibouti; 2 Laboratory of Analytical and Molecular Chemistry, Hassan II University of Casablanca, Casablanca 20100, Morocco; 3 Peltier Hospital of Djibouti, Djibouti City 77101, Djibouti; 4 Independent Researcher, E-46022 Valencia, Spain

**Keywords:** malaria, *Plasmodium falciparum*, *Plasmodium vivax*, medicinal plants, molecular docking, ADMET, molecular dynamics, drug discovery, in silico study

## Abstract

Objectives: Malaria remains a major global health burden, particularly in endemic regions such as Djibouti, where *Plasmodium falciparum* and *Plasmodium vivax* co-circulate, complicating disease control strategies. Increasing resistance to current antimalarial drugs reduces treatment effectiveness and highlights the urgent need for new, safe, and affordable therapeutic agents. This study aimed to identify potential inhibitors from Djiboutian medicinal plants using an integrated in silico approach targeting key proteins from both parasite species. Methods: A library of 222 phytoconstituents was screened against *Plasmodium vivax FK506-binding protein 35* (PDB ID: 3IHZ) and *Plasmodium vivax* dihydrofolate reductase–thymidylate synthase (PDB ID: 1J3K) using molecular docking. Top-ranked compounds were further analyzed for binding interactions and evaluated for drug-likeness and pharmacokinetic properties using QikProp in Maestro v11.5. Selected protein–ligand complexes were subjected to 100 ns molecular dynamics simulations, and their stability was assessed using multiple descriptors, including structural deviation, flexibility, compactness, solvent exposure, and hydrogen bond persistence. Results: Several phytoconstituents exhibited strong binding affinities, with docking scores ranging from −6.09 to −7.54 kcal/mol, outperforming the reference drug artemisinin. Interaction analysis revealed key hydrogen bonds and hydrophobic contacts with essential active-site residues. ADMET predictions indicated favorable pharmacokinetic profiles, including high oral absorption, good membrane permeability, and low predicted toxicity. Molecular dynamics simulations demonstrated stable behavior for most complexes, with compound 121 showing enhanced stability in the 1J3K system and compound 123 exhibiting consistent dynamic stability in the 3IHZ system. In contrast, compound 82 displayed greater structural fluctuations despite maintaining stable hydrogen bond interactions. Conclusions: The integration of molecular docking, ADMET prediction, and molecular dynamics simulations identified compounds 121 and 123 as the most promising antimalarial candidates, exhibiting an optimal balance of binding affinity, favorable pharmacokinetic properties, and dynamic stability. These findings highlight the potential of Djiboutian medicinal plants as a valuable source of novel antimalarial agents and provide a strong computational foundation for future experimental validation.

## 1. Introduction

Malaria remains one of the most significant global public health challenges, particularly in Sub-Saharan Africa, where the majority of cases and deaths occur. In 2023, an estimated 263 million cases and nearly 600,000 deaths were reported worldwide, with approximately 94% of deaths occurring in Africa [1]. Malaria is caused by parasites of the genus *Plasmodium*, transmitted through the bites of infected female *Anopheles* mosquitoes. Among the five species infecting humans, *Plasmodium falciparum* and *Plasmodium vivax* are the most clinically significant and epidemiologically important [2].

In Djibouti, the epidemiology of malaria is complex due to the co-circulation of these two predominant parasite species. Recent data indicate that approximately 77% of infections are attributed to *P. falciparum*, while *P. vivax* accounts for roughly 33% of cases [2,3]. The coexistence of these species leads to unique transmission dynamics, as they differ biologically, clinically, and in their response to control measures. Over the past decade, malaria incidence in Djibouti has significantly increased following the introduction of the invasive vector *Anopheles stephensi* in 2012 [4]. The country, which was previously close to malaria elimination, has experienced a dramatic resurgence of the disease. Reported malaria cases increased from fewer than 2000 in 2013 to nearly 39,000 in 2023 [5]. More than 90% of these cases were reported in urban areas, particularly in Djibouti City [6,7].

Molecular studies have detected *P. vivax* sporozoites in field-collected mosquitoes, confirming active transmission cycles. This finding highlights the role of the invasive vector in maintaining malaria transmission in urban settings. The interaction between *P. falciparum* and *P. vivax* further complicates malaria transmission in Djibouti. Mathematical modeling studies have demonstrated that co-infection dynamics play a crucial role in disease spread and persistence [2,8,9]. The basic reproduction numbers of *Plasmodium falciparum* (R_0_f) and *Plasmodium vivax* (R_0_v) are important indicators of transmission potential. The overall disease dynamics are largely determined by the higher of these two values. These findings emphasize the importance of considering both species simultaneously when designing effective control strategies [2,8,9].

While *Plasmodium falciparum* is generally associated with severe disease and higher mortality rates, *Plasmodium vivax* presents additional challenges. This species can form dormant liver stages, known as hypnozoites, which may reactivate weeks or months after the initial infection and cause relapses [10]. This biological feature complicates treatment and contributes to sustained transmission, even in areas with effective control measures. Furthermore, *P. vivax* infections are often characterized by lower parasitemia levels. As a result, they can be more difficult to detect using standard diagnostic methods [11]. Malaria transmission is also influenced by seasonal and environmental factors. Peak transmission is typically observed between March and August, corresponding to favorable conditions for mosquito breeding [6,12]. Urbanization also plays an important role. Water storage practices and population movement further contribute to sustained transmission. This is particularly evident in densely populated areas [6,12].

At the same time, diagnostic challenges complicate malaria control efforts. Rapid diagnostic tests (RDTs), especially those based on histidine-rich protein 2 (HRP2), are widely used for detecting *P. falciparum* infections [7,13]. However, the emergence of *pfhrp2/3* gene deletions has significantly reduced test sensitivity. This leads to false-negative results and contributes to underdiagnosis [13,14,15]. This is particularly problematic in regions where both *P. falciparum* and *P. vivax* coexist, as misdiagnosis may result in inappropriate treatment and continued transmission.

Moreover, recent epidemiological and modeling studies have shown that malaria transmission in Djibouti is influenced by several factors, including mosquito biting rates, vector density, and environmental conditions [6]. Sensitivity analyses indicate that parameters such as biting rate and transmission probability play a critical role in sustaining the circulation of both parasite species [2]. When the basic reproduction number exceeds one (R_0_ > 1), the infection persists in the population. In contrast, reducing R_0_ below one may lead to disease elimination [2].

The coexistence of *P. falciparum* and *P. vivax*, combined with vector adaptation and diagnostic limitations, makes malaria control in Djibouti particularly challenging. Conventional strategies, such as insecticide-treated nets (ITNs) and indoor residual spraying (IRS), may be insufficient due to changes in vector behavior and increasing insecticide resistance [4]. Additionally, the presence of relapsing *P. vivax* infections and undetected *P. falciparum* cases further complicates elimination efforts.

Improving malaria control strategies requires a better understanding of the transmission dynamics of both parasite species. Enhancing genomic surveillance, improving diagnostic accuracy, and integrating predictive modeling tools are essential steps toward reducing disease burden and achieving long-term elimination goals in Djibouti. In this context, the present study aims to explore safer, more accessible, and locally available therapeutic options for malaria. Although current treatments are effective, they may be associated with high costs, side effects, and limited accessibility in endemic regions such as Djibouti. Furthermore, the emergence of drug resistance highlights the urgent need for alternative and sustainable therapeutic strategies.

Medicinal plants, widely used in traditional medicine, represent a promising source of bioactive compounds with potential antimalarial activity. This selection is based on ethnobotanical knowledge. The study focuses on plant species known for their antimicrobial, anti-inflammatory, and antiparasitic properties. It aims to evaluate their effectiveness against *Plasmodium* parasites, particularly *P. falciparum* and *P. vivax*. By scientifically investigating these plants, the study seeks to identify novel, safe, and affordable therapeutic candidates. These candidates could contribute to improved malaria control in endemic regions.

## 2. Material and Methods

### 2.1. Data Sources

The dataset consisted of 222 phytoconstituents experimentally isolated and identified from medicinal plants native to Djibouti, as reported in published phytochemical studies [16]. The selected plant species were chosen based on ethnobotanical surveys documenting their traditional use in the management of malaria, febrile illnesses, and other infectious diseases. Although these phytoconstituents have previously been characterized for various biological activities, their potential antimalarial activity has not yet been comprehensively investigated. Therefore, this library was selected to systematically evaluate its inhibitory potential against key *Plasmodium falciparum* and *Plasmodium vivax* targets using an integrated in silico approach. The 2D and 3D structures of the selected compounds were generated using ChemOffice 19.0 software. Table S1 (Supplementary Materials) provides detailed information on the studied compounds, including plant name, compound name, SMILES notation, and region of origin.

### 2.2. Molecular Docking Studies

Molecular docking predicts (with a high degree of accuracy) the optimal binding of a ligand to the enzyme’s active site by providing essential information on intermolecular interactions between a ligand and its target protein as well as the interaction’s binding free energy and stability of the protein–ligand complex. This study utilized a library comprising 222 phytoconstituents isolated from medicinal plants in Djibouti, which were docked against two validated antimalarial targets, Plasmodium falciparum dihydrofolate reductase–thymidylate synthase (1J3K) and Plasmodium vivax FK506-binding protein 35 (3IHZ), using Schrödinger Maestro v11.5 following a well-defined computational protocol. Artemisinin was selected as the reference compound because it is the cornerstone of current antimalarial therapy and serves as a well-established standard for evaluating the binding affinity of potential antimalarial candidates. Comparing the docking performance of the screened phytoconstituents with that of artemisinin provided a benchmark for assessing their predicted inhibitory potential against the selected malaria targets.

#### 2.2.1. Preparation of Ligands

The molecules were prepared for molecular docking calculations using the LigPrep 2.0 tool, integrated into version 11.5 of the Schrödinger Maestro v11.5 software. The preparation was carried out using the OPLS3 force field, with the generation of ionization states corresponding to pH values of 7.0 and 2.0. When undefined chiral centers were present, LigPrep generated up to 32 possible stereoisomers for each ligand to ensure that all plausible stereochemical configurations were considered during docking. Using the same approach, artemisinin was prepared as a reference compound. Its structure was downloaded from the PubChem database.

#### 2.2.2. Malaria Therapeutic Targets Selection

Target selection is a critical step in structure-based drug discovery. In the present study, two validated antimalarial targets, 1J3K and 3IHZ, were selected because they play essential roles in parasite survival and have been recognized as promising therapeutic targets [17,18,19]. The 1J3K structure corresponds to the quadruple mutant Plasmodium falciparum dihydrofolate reductase–thymidylate synthase (PfDHFR–TS), a key enzyme involved in folate metabolism and DNA synthesis. Inhibition of PfDHFR–TS disrupts nucleotide biosynthesis, thereby preventing parasite replication. Moreover, mutations in this enzyme are strongly associated with resistance to antifolate drugs such as pyrimethamine, making it an important target for antimalarial drug development [17,18]. The 3IHZ structure corresponds to the FK506-binding protein 35 (FKBP35) of Plasmodium vivax, a peptidyl-prolyl cis–trans isomerase involved in protein folding and parasite survival. Previous studies have demonstrated that FKBP35 is a promising antimalarial target because compounds interacting with this protein can interfere with essential cellular processes in the parasite [18,19].

#### 2.2.3. Protein Preparation

The crystal structures of the two target proteins (1J3K and 3IHZ) were downloaded from the Protein Data Bank (PDB) [20,21]. The protein was prepared using the Protein Preparation Wizard tool in Schrödinger Maestro v11.5 software [22]. This step included the removal of water molecules, the addition of hydrogen atoms to heavy atoms, and the appropriate assignment of protonation states. Energy minimization was then performed using the OPLS3 force field, with a maximum RMSD value of 0.30 Å for heavy atoms.

#### 2.2.4. Grid Box Coordinates

The receptor grid for proteins 1J3K and 3IHZ was generated by centering the grid box on the co-crystallized ligand present in each protein structure, thereby defining the active binding site, using the Receptor Grid Generation tool (Schrödinger Maestro v11.5). A cubic grid box with dimensions of 20 × 20 × 20 Å^3^ was used to fully encompass the ligand-binding pocket while providing sufficient space for ligand sampling during the docking process. Three docking protocols were used to evaluate potential ligands: high-throughput virtual screening (HTVS), standard precision (SP), and enhanced precision (XP). This hierarchical protocol was applied progressively, starting with HTVS mode, followed by SP mode, and then XP mode to optimize the accuracy of poses and binding scores [23,24,25].

### 2.3. Drug-Likeness and ADMET Prediction

The evaluation of the pharmacokinetic properties of candidate compounds is an important aspect in determining whether a compound can be therapeutically active. For this study, the pharmacokinetic properties were predicted using the QikProp module found in Schrödinger Maestro v11.5 to help estimate the physicochemical and pharmacokinetic characteristics important to the design of drug compounds and oral bioavailability. According to the QikProp guidelines, acceptable QPlogBB values typically range from −3.0 to 1.2, where higher values indicate greater predicted blood–brain barrier permeability, whereas lower values suggest limited penetration into the central nervous system. Candidate compounds were also tested for compliance against Lipinski’s Rule of Five. This includes molecular weight, lipophilicity (QPlog Po/w), the number of hydrogen bond donors, the number of hydrogen bond acceptors, and several desired pharmacokinetic parameters based on QikProp predictions, including aqueous solubility (QPlog S), Caco-2 cell permeability (QPPCaco), blood–brain barrier penetration (QPlog BB), and absorption in human beings via oral administration. The results from these analyses as predicted by QikProp can provide critical insight into how the candidate compounds would be absorbed and distributed in humans and their potential bioavailability. The rapid screening of most candidate molecules using QikProp allows for the efficient identification of candidate molecules with the desired pharmacokinetic and acceptable safety profiles that are ready to progress into the next phase of drug development [23,24,25].

### 2.4. Molecular Dynamics Simulation

To evaluate the structural stability of the best protein–ligand complexes identified by molecular docking, 100 ns molecular dynamics (MD) simulations were performed using the Desmond module of Schrödinger Maestro v11.5. The systems to be modeled were prepared by removing overlapping water molecules and optimizing the structure. Each protein–ligand complex was solvated using the TIP3P water model with a 10 Å buffered distance and periodic box boundaries. The structure was neutralized by use of Na+ or Cl- ions at a physiologically relevant ionic strength of 0.15 M. The OPLS3e forcefield was used followed by the minimization of energy up to a 100 ns simulation time for each protein–ligand complex at 300 K and 1.01 atm pressure with Nose–Hoover and Martyna-Tobias-Klein thermostat/barostat systems (NVT/NPT ensembles) [26,27]. Each of the production runs was performed over 100 ns and all production trajectory data were recorded every 100 ps. The structural stability of the complexes was identified through root mean square deviation (RMSD) values from the starting structure, root mean square fluctuation (RMSF) values, and hydrogen bonding analysis.

### 2.5. MM-GBSA Calculation

The binding free energies (ΔG_binding) of the protein–ligand complexes were calculated using the Prime MM/GBSA module implemented in Schrödinger Maestro v11.5. The calculations were performed using the final representative structure (last frame) extracted from the 100 ns molecular dynamics trajectory for each complex. The binding free energy was estimated according to the equation ΔG_binding = G_complex − (G_protein + G_ligand), where G_complex, G_protein, and G_ligand represent the free energies of the complex, the isolated protein, and the isolated ligand, respectively. The MM/GBSA approach accounts for molecular mechanics and implicit solvation effects, providing a more reliable estimation of ligand binding affinity than docking scores alone [28].

## 3. Results and Discussion

### 3.1. Docking Analysis

The molecular docking technique aims to predict the optimal position of a ligand within the binding site of a receptor and to accurately estimate the associated binding affinity. In this study, two docking approaches, namely rigid docking and flexible docking, were applied to compare their performance in predicting ligand–protein interactions. In the rigid docking protocol, the receptor structure was kept fixed during docking, whereas in the flexible docking protocol, ligand conformational flexibility was considered to allow for improved accommodation within the binding pocket. Comparing both approaches enabled the evaluation of the influence of ligand flexibility on the predicted binding affinity and interaction patterns. The binding affinities and best binding poses of the molecules were evaluated and compared with those of the reference drug artemisinin after docking into the active sites of the 1J3K and 3IHZ proteins. The docking scores obtained from both docking modes for the studied molecules and the reference compound are presented in Table 1 and Table 2.

The results of Table 1 showed that molecules 11, 121, and 9 had higher binding affinities to the 1J3K protein than artemisinin. Both in flexible and rigid mode, molecules 11 and 9 had similar interaction profiles (hydrogen bonding and hydrophobic interactions), indicating a stable binding within the active site of the 1J3K protein. A significant decrease in docking score in the rigid mode for molecule 121 shows that flexibility promotes its binding with the active site. Molecule 118 showed the lowest docking score (−4.35 and −4.64 Kcal/mol) in both protocols. These results show that docking with flexible ligands produces slightly better binding scores. The Glide e-model parameter was used to select the most favorable binding pose for each ligand among the generated docking conformations. These results show that these four molecules have greater inhibitory activity than artemisinin.

The docking results against the 3IHZ protein (Table 2) revealed that molecules 123 and 132 exhibited the highest binding affinities in both flexible and rigid docking modes, with GlideScores close to −6.8 kcal/mol, significantly better than artemisinin (≈−4.9 kcal/mol). Both compounds formed a key hydrogen bond with ILE74 and were further stabilized by hydrophobic π–alkyl and π–sigma interactions. Molecule H1 showed moderate affinity, whereas molecule 82 displayed the lowest affinity among the tested compounds and presented an unfavorable donor–donor interaction with TYR43. The similarity of the docking scores and interaction patterns between the flexible and rigid protocols indicates that the binding modes of these ligands are largely preserved regardless of protein flexibility.

### 3.2. Pharmacokinetic and Toxicity Profile Prediction

After conducting molecular docking, subsequent analysis of the pharmacokinetic properties of the top-ranked compounds in terms of ADMET (Absorption, Distribution, Metabolism, Excretion, and Toxicity) was conducted to obtain a comprehensive and well-rounded assessment of their potential in the treatment of a given disease/condition. The results of this analysis are provided in Table 3 and show that each of these compounds has a very similar pharmacokinetic profile, with respect to their ability to bind/attach to the 1J3K Protein.

The predicted ADMET properties of the selected ligands against the 3IHZ target are summarized in Table 3. All investigated compounds complied with Lipinski’s Rule of Five, exhibiting no violations and therefore demonstrating favorable drug-like characteristics and oral bioavailability potential. All selected compounds exhibited QPlogBB values within the acceptable range predicted by QikProp (−3.0 to 1.2), suggesting suitable blood–brain barrier permeability. Compounds 82, 132, and 123 displayed balanced lipophilicity and solubility profiles, with QPlogPo/w values ranging from 1.16 to 2.25 and QPlogS values indicating acceptable aqueous solubility. High predicted Caco-2 permeability values were observed for all compounds, suggesting efficient intestinal membrane permeability and absorption, comparable to or exceeding that of the reference drug artemisinin. Oral absorption values remained high (≥94%), further supporting their suitability for oral administration. Additionally, QPlogBB predictions indicated acceptable blood–brain barrier penetration, while HERG inhibition values suggested manageable cardiotoxicity risk. Overall, the selected ligands demonstrated favorable pharmacokinetic profiles against the 3IHZ target, supporting their further investigation as potential antimalarial candidates.

The predicted ADMET profiles of the selected ligands against the 1J3K target are presented in Table 4. All compounds complied with Lipinski’s Rule of Five and demonstrated favorable drug-like characteristics. QPlog Po/w values within the recommended range indicate appropriate lipophilicity for drug candidates, whereas QPlogBB values provide an estimate of blood–brain barrier permeability, with lower values generally suggesting limited penetration into the central nervous system. Their lipophilicity values (QPlogPo/w ≈ 3.79–4.02) suggested enhanced membrane permeability, although the relatively low aqueous solubility values may indicate a permeability–solubility trade-off. Nevertheless, all compounds exhibited exceptionally high Caco-2 permeability compared with the reference drug artemisinin, indicating strong intestinal absorption potential. Compound 118 displayed the highest predicted permeability, whereas compounds 121 and 11 maintained a more balanced pharmacokinetic profile through the combination of favorable permeability and physicochemical properties. Oral absorption values reached 100% for all tested ligands, and QPlogBB values remained within the acceptable limits, suggesting controlled central nervous system exposure. Furthermore, HERG inhibition predictions suggested a relatively low cardiotoxicity risk. Overall, these findings support the favorable pharmacokinetic suitability of compounds targeting 1J3K and justify their selection for subsequent computational investigations.

Although artemisinin exhibited limited hydrogen-bond interactions with key residues in the present docking analyses, its antimalarial activity is known to involve mechanisms beyond conventional ligand–receptor hydrogen bonding. Artemisinin contains a characteristic endoperoxide bridge that becomes activated in the presence of ferrous iron (Fe^2+^) or heme released during hemoglobin digestion within the malaria parasite. This activation generates highly reactive radical intermediates and reactive oxygen species capable of damaging parasite proteins, membranes, and essential metabolic pathways. Consequently, the pharmacological activity of artemisinin is primarily associated with oxidative damage and multi-target effects rather than dependence on strong hydrogen-bond interactions with a single protein target. This mechanism may explain why artemisinin remains an effective antimalarial reference despite showing limited hydrogen-bond formation in the present computational analyses.

### 3.3. Binding Interaction Analysis of Selected Compounds

**Figure 1:**
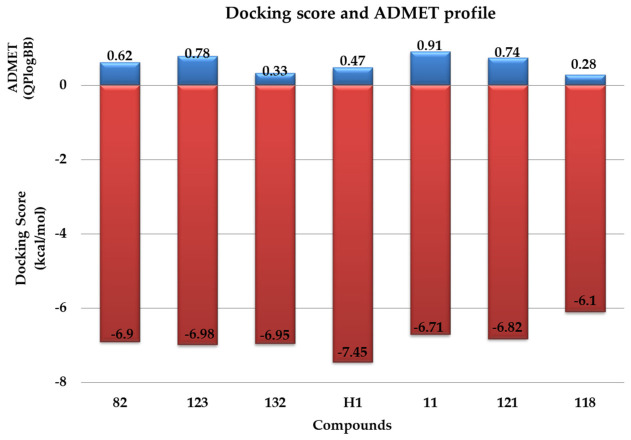
Combined evaluation of docking affinity and ADMET profile of the selected compounds.

The combined evaluation of docking affinity and ADMET profile (Figure 1) highlights compounds 82 and 123 as the most promising candidates, exhibiting an optimal balance between binding strength and pharmacokinetic properties. In contrast, compounds such as H1, despite showing strong binding affinity, were excluded due to unfavorable ADMET characteristics.

**Figure 2:**
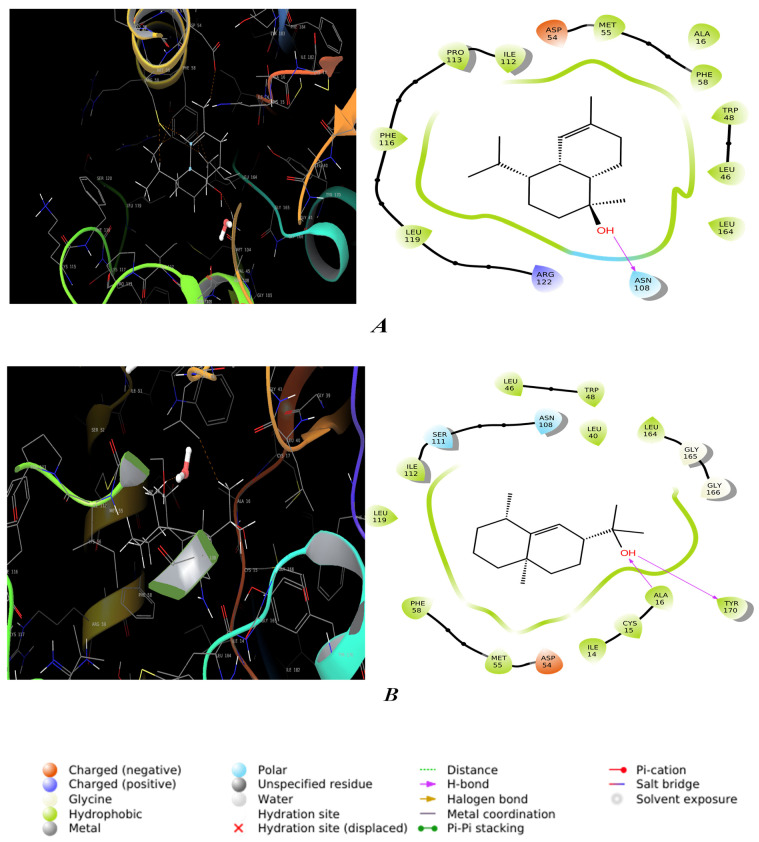
Binding interaction analysis of selected compounds with the 1J3K protein. (**A**) Binding mode of compound 11 within the active site. (**B**) Interaction profile of compound 121 showing hydrogen bonding and hydrophobic interactions.

**Figure 3:**
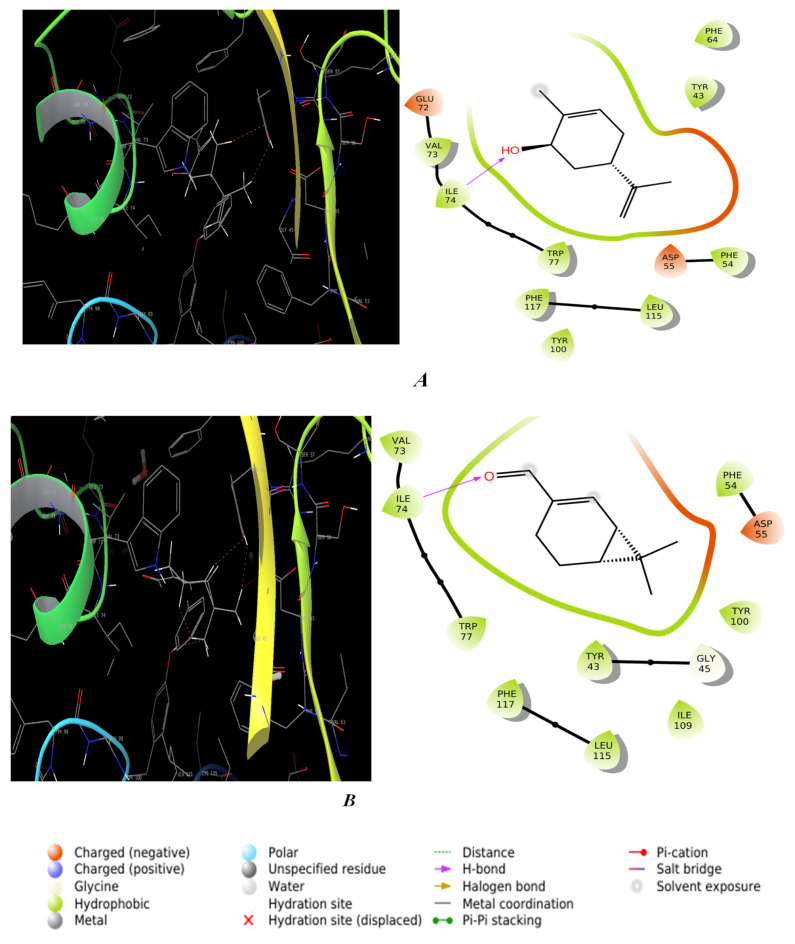
Binding interaction analysis of selected compounds with the 3IHZ protein. (**A**) Binding mode of compound 82 within the active site. (**B**) Interaction profile of compound 123 highlighting hydrogen bonding and hydrophobic interactions with key residues.

To gain deeper insights into the molecular determinants of binding, a comprehensive interaction analysis was performed, focusing on hydrogen bonding, hydrophobic contacts, and key residue contributions within the active site. The binding modes and interaction patterns of the selected compounds are illustrated in Figure 2 and Figure 3.

The binding interaction analysis of compound 11 and compound 121 in the binding pocket region of the 1J3K protein revealed distinct and specific interaction patterns that stabilize each ligand to the 1J3K protein. An example of the interaction profile for compound 11 is located in Figure 2A. As evident in Figure 2A, compound 11 occupies an appropriate interior location in the binding pocket and establishes hydrogen bonding interactions via its hydroxyl functional group to the ASN108 residue, which will assist in anchoring compound 11 in the binding pocket of the 1J3K protein. Furthermore, compound 11 forms a hydrophobic environment with respect to the following residues: PHE58, LEU119, ILE112, and PRO113. These hydrophobic contacts reinforce the stability of the binding interaction between compound 11 and the 1J3K protein.

The following residues, TRP48 and LEU46, enhance the stability of compound 11 through the establishment of van der Waals interactions. In comparison, Figure 2B illustrates the interaction profile of compound 121 with the 1J3K protein. Compound 121’s binding is largely stabilized through several hydrophobic contacts including LEU164, LEU46, ILE112, and PHE58. Collectively, these hydrophobic contacts contribute to the anchoring of compound 121 in the binding pocket of the 1J3K protein. Additionally, the hydroxyl functional group of compound 121 forms a hydrogen bond with the TYR170 residue, further stabilizing compound 121 in the binding pocket. There is also evidence of polar contacts via several residues (ASN108 and SER111) that contribute to the overall positioning of compound 121. Overall, both compounds exhibit stable binding within the 1J3K active site through a combination of hydrogen bonding and hydrophobic interactions. However, compound 11 shows a slightly more favorable interaction profile due to its stronger polar interaction with ASN108, whereas compound 121 relies more on hydrophobic stabilization. These findings are consistent with the docking results and support the selection of these compounds for further dynamic analysis.

The interaction analysis associated with the binding pocket of the 3IHZ ligands (Figure 3) revealed more about the stabilization modes of the ligands we selected. As shown in Figure 3A, compound 82 resides deep inside the active site of the enzyme, with its hydroxyl group making a critical hydrogen bond with ILE74. This bond suggests that the hydroxyl group plays an important role in anchoring the ligand to the active site. The surrounding environment is primarily hydrophobic, with the creation of a more compact binding pocket due to the presence of TRP77, PHE117, VAL73, and LEU115, increasing the retention of the ligand through van der Waals interactions. In addition, the relationship between the hydrogen bond formed between the ligand and ILE74 and the proximity of TYR100 and ASP55 further suggest that the ligand may be stabilized by complementary interactions with these surrounding residues. Alternatively, while compound 123 adopts a similar orientation as compound 82 and has a preserved hydrogen bond to ILE74 (Figure 3B), its mode of stabilization is mainly hydrogen bond and hydrophobic interactions with TRP77, PHE117, LEU115, and TYR43. The predominance of hydrophobic interactions also suggests that compared to compound 82, compound 123 relies more heavily on non-polar interactions to maintain its position within the active site. Both VAL73 and ASP55 provide additional structural complementarity between the ligand and the protein cavity, adding to the stabilizing characteristics of the ligand. Taken together, both ligands display favorable accommodation within the 3IHZ active site, with a shared anchoring interaction and a complementary hydrophobic environment. These interaction features agree with their predicted binding affinities and further support their potential as promising candidates for subsequent molecular dynamics studies. While docking and ADMET analyses provide valuable insights into binding affinity and pharmacokinetic behavior, they do not account for the dynamic nature of protein–ligand interactions. Therefore, molecular dynamics simulations were conducted to further evaluate the stability and behavior of the selected complexes under physiological conditions over time.

### 3.4. Molecular Dynamics Simulation Analysis

Based on docking and pharmacokinetic screening, two promising ligands were selected for each target protein for further molecular dynamics investigations. Compounds 11 and 121 were selected for the 1J3K system, whereas compounds 82 and 123 were retained for the 3IHZ system. To further validate the stability and dynamic behavior of the selected protein–ligand complexes, molecular dynamics (MD) simulations were performed. The trajectories were analyzed using several key parameters, including root mean square deviation (RMSD), root mean square fluctuation (RMSF), radius of gyration (Rg), solvent accessible surface area (SASA), polar surface area (PSA), and hydrogen bond interactions. These analyses provide comprehensive insights into the structural stability, flexibility, and interaction persistence of the complexes over the simulation time.

#### 3.4.1. Root Mean Square Deviation Analysis

**Figure 4:**
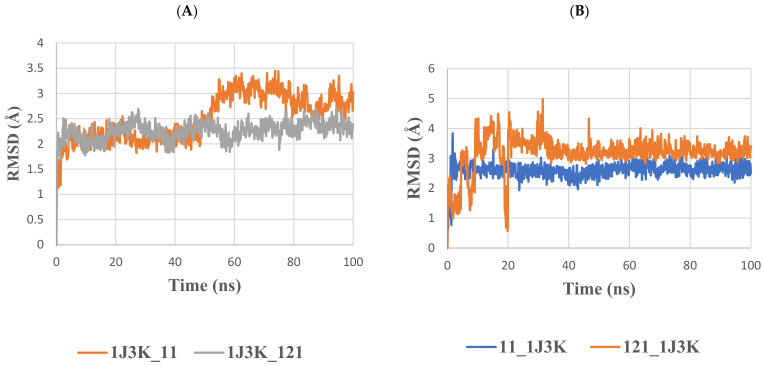
Protein RMSD (**A**) and ligand RMSD (**B**) of the complexes 11–1J3K and 121–1J3K.

Direct insight into the stability of protein–ligand complexes during their entire simulation duration was obtained from monitoring the root mean square deviation (RMSD) of each complex. Protein–ligand complexes undergo an initial equilibration phase, followed by a stable RMSD, indicating successful achievement of equilibrium. In addition to this, ligands binding to proteins produce minimal protein conformational deviation, which provides additional stability to proteins overall. Figure 4 shows the detailed RMSD analysis of the 1J3K protein–ligand complexes, including both the protein and ligand RMSDs for both complexes during their entire simulation durations. Figure 4A,B shows that after the initial equilibration phase, there was no significant deviation of the protein of either complex beyond approximately 2.0–2.5 Å (overall), indicating that both complexes maintain their global stability, while subjecting the protein to both ligands does not produce any significant conformational change to the protein. However, compound 121 displays lower fluctuation than compound 11 suggesting that compound 121 provides a slightly higher level of stability to the protein backbone relative to compound 11. Overall, the RMSD results confirm that both complexes exhibit stable dynamic behavior, with compound 121 contributing slightly more to protein stabilization, while compound 11 maintains a more consistent ligand conformation within the binding site.

**Figure 5:**
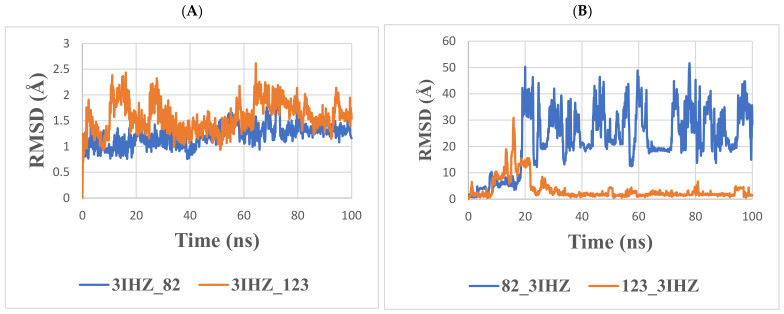
Protein RMSD (**A**) and ligand RMSD (**B**) of the complexes 82–3IHZ and 123–3IHZ.

The root mean square deviation analysis of the 3IHZ complexes (Figure 5) provides insight into the structural stability of both the protein and ligand systems. As shown in Figure 5A, the protein RMSD profiles remained relatively stable throughout the simulation, fluctuating within a narrow range of approximately 1.0–1.8 Å. This behavior indicates that the protein structure maintains its integrity upon ligand binding. Notably, the complex with compound 82 exhibited slightly lower fluctuations compared to compound 123, suggesting enhanced stabilization of the protein backbone.

In contrast, the ligand RMSD profiles (Figure 5B) revealed distinct behaviors between the two compounds. Compound 123 maintained low and stable RMSD values, indicating a consistent binding mode within the active site. However, compound 82 displayed significantly higher fluctuations, which may indicate substantial conformational changes or displacement within the binding pocket during the simulation. Therefore, the stability of compound 123 appears to be more favorable in terms of maintaining a consistent binding orientation.

#### 3.4.2. Root Mean Square Fluctuation Analysis

**Figure 6:**
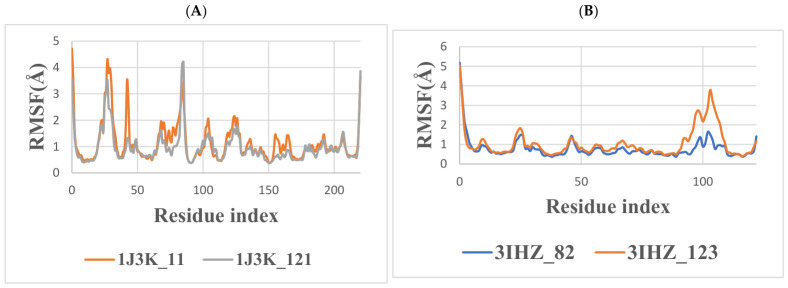
Root mean square fluctuation analysis of (**A**) 1J3K–11 and 1J3K–121 and (**B**) 3IHZ–82 and 3IHZ–123.

Root mean square fluctuation analysis can be used to assess how flexible a particular individual amino acid residue is. The results show that in most proteins, the fluctuations in their respective regions compared to their own are mainly located in the loop regions whereas some residues in the binding site are held more rigid. This means that the binding of a ligand will reduce the amount of local motion and improve the stability of areas that are critical to the function of the protein. Root mean square fluctuation (Figure 6) analysis provides information on the flexibility of residues of the amino acids in both proteins. The 1J3K complexes (shown in Figure 6A) had similar fluctuation patterns between themselves and had low RMSF values (fluctuation measures) on almost all residues, indicating a relatively stable protein structure. Also, it can be noted that higher fluctuations were primarily located near the termini and loop regions, which tend to have more flexibility than other areas in the protein. The fluctuations for compound 121 were slightly less than those for compound 11, hence compound 121 had a slightly greater stabilizing effect on the protein backbone. As shown in Figure 6B, the 3IHZ complexes also had low RMSF values, providing evidence of stable structures during the course of the simulations, however, some fluctuations in the residues for compound 123 were present in parts of the protein, i.e., in the area of residues 90–110, indicating a localized movement of the residue in that specific region. In addition, while compound 82 had a uniform and stable RMSF pattern over the complete length of the protein, both systems maintained a relatively stable RMSF pattern for residues that were located within their respective binding sites, indicating that the ligand–protein interaction remained intact.

#### 3.4.3. Radius of Gyration Analysis

**Figure 7:**
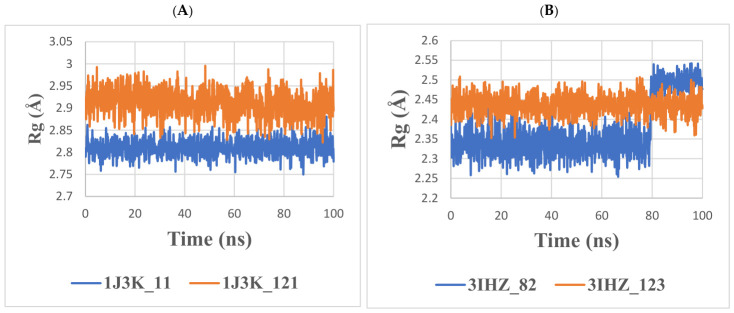
Radius of gyration analysis of (**A**) 1J3K–11 and 1J3K–121 and (**B**) 3IHZ–82 and 3IHZ–123.

The compactness of protein structures was evaluated through their radius of gyration during the course of the simulations performed. The stability of this parameter throughout the trajectory suggests that there is no significant unfolding or conformational rearrangement due to ligand binding, and therefore, the proteins are structurally stable. Moreover, the radius of gyration profiles (Figure 7) demonstrate insights regarding protein compactness throughout each simulation. For example, for the 1J3K system (Figure 7A), the Rg values for both complexes remained essentially unchanged throughout the trajectory, indicating that the global fold of both proteins was preserved. However, the complex with compound 11 had slightly lower Rg value than the complex with compound 121, which suggests that the former has a more compact structural arrangement than the latter. Similarly, for the 3IHZ system (Figure 7B), the Rg values also remained within a very small range, indicating that there were stable structural deviations throughout the entire trajectory. In the case of the compound 123, the Rg profile remained consistent throughout the entire trajectory, whereas for compound 82, the Rg values gradually increased after approximately 80 ns, potentially due to slight conformational adjustments.

Regardless of this minor deviation from the expected values, there were no major structural disruptions as a consequence of ligand binding in either protein, which indicates that both the ligand bound proteins maintain their structural integrity with relatively low levels of variation in terms of protein compactness during the trajectory of both simulations.

#### 3.4.4. Solvent Accessible Surface Area Analysis

**Figure 8:**
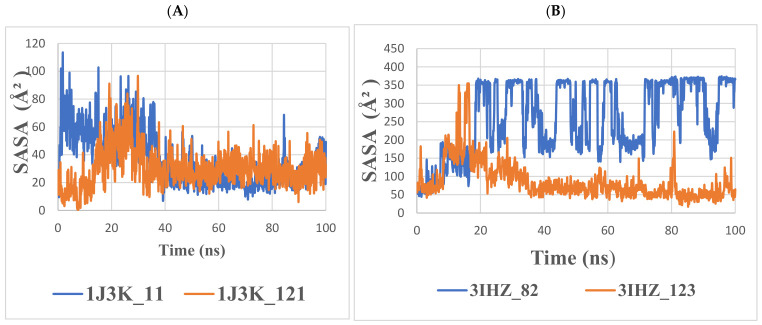
Solvent accessible surface area analysis of (**A**) 1J3K–11 and 1J3K–121 and (**B**) 3IHZ–82 and 3IHZ–123.

The solvent-accessible surface area was measured to determine how much of the protein’s surface is accessible to the solvent. The values showed little fluctuation over the course of the simulation, indicating that binding to the ligands did not substantially alter the accessibility of the protein surface to the solvent. The slight fluctuations may indicate that there was some amount of subtle conformational changes occurring in the protein. The profile of each complex in the SASA (Figure 8) provides information on how the accessibility of the protein surface to the solvent changes over the course of the simulation. For the 1J3K system (Figure 8A), both complexes had decreased SASA values in the initial phase, and stabilized at lower SASA values for the remainder of the simulation. This pattern indicates that there was a structural adjustment that made the protein more compact and therefore had less surface exposure to the solvent. Additionally, the two complexes behaved in a similar manner; therefore, ligand binding for both compounds appears to have caused similar effects on the protein surfaces. On the contrary, the 3IHZ system (Figure 8B) showed different behaviors between the two ligands. Compound 123 SASA values were consistently low throughout the simulation (i.e., compound 123 retained a compact structure with limited surface exposure). Conversely, compound 82 SASA values were significantly higher for the entire simulation and showed considerable fluctuation; both of which suggest increased surface exposure and probable conformational changes. This behavior may indicate a less stable structural adaptation in comparison to compound 123.

These observations highlight that while both ligands maintain stable interactions in the 1J3K system, compound 123 appears to promote a more compact and less solvent-exposed conformation in the 3IHZ system.

#### 3.4.5. Polar Surface Area Analysis

**Figure 9:**
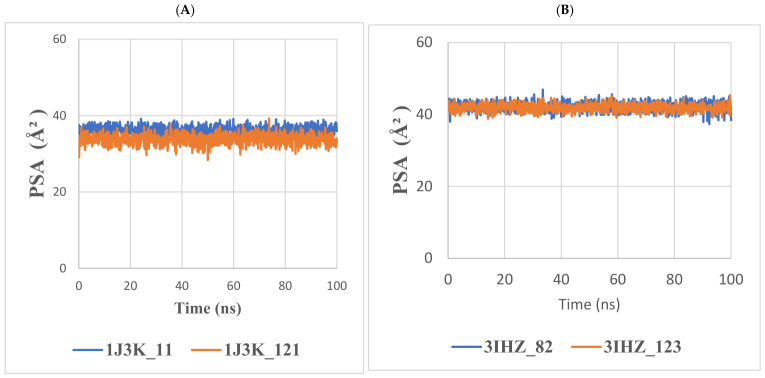
Polar surface area analysis of (**A**) 1J3K–11 and 1J3K–121 and (**B**) 3IHZ–82 and 3IHZ–123.

The analysis of the polar surface area shows how polar interactions behave during simulation. The values remained constant throughout the simulation, which suggests that the ligand is consistently in contact with the protein residues and therefore there is a well-established network of stable interactions within the binding site. The polar surface area profiles (Figure 9) were also very consistent from start to finish for both protein systems. In the 1J3K complexes (Figure 9A), minor fluctuations occurred within a narrow range, which shows that the system’s characteristics remained intact throughout the duration of the simulation. A small difference existed between the compounds’ overall appearance; however, both compounds provided a similar contribution to the long-term stability of the polar interactions that are formed when a ligand binds to a protein. The same trend was seen for the 3IHZ system (Figure 9B) where both complexes had stable polar surface area values with little variation. This means that throughout the duration of the simulation, polar contacts remained intact between the ligand and its binding partner; therefore, the overall number of polar areas within the three-dimensional shape of the protein structure will not change significantly when the ligand binds to it. The stability of the system’s polar surface area indicates that the network of polar interactions was very stable throughout the simulation, and subsequently supports the overall consistency of protein–ligand complex structures.

#### 3.4.6. Hydrogen Bond Analysis

**Figure 10:**
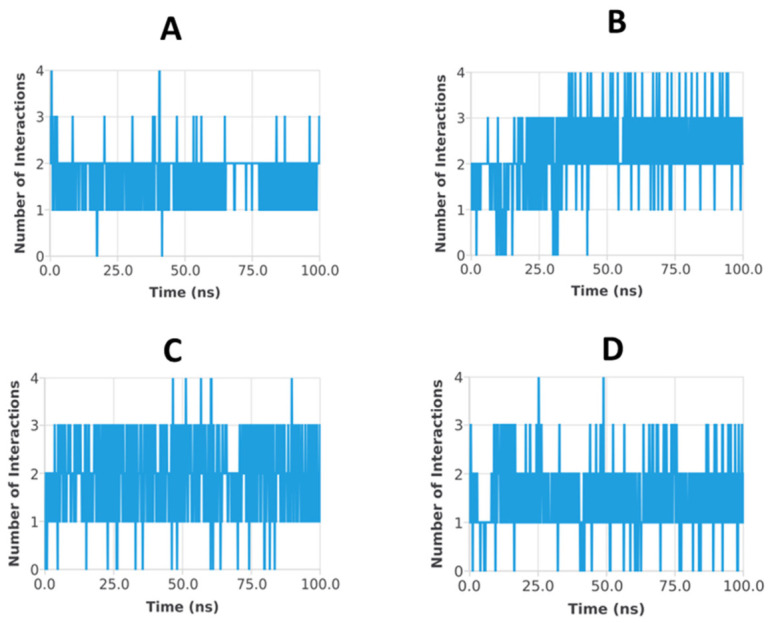
Hydrogen bond analysis of the complexes (**A**) 1J3K–11, (**B**) 1J3K–121, (**C**) 3IHZ–82, and (**D**) 3IHZ–123.

Hydrogen bond analysis was performed to examine the persistence of key intermolecular interactions throughout the simulation. The relatively stable number of hydrogen bonds observed suggests strong and sustained interactions between the ligands and the protein, contributing significantly to the stability of the complexes. The hydrogen bond analysis (Figure 10) provides valuable insight into the persistence and stability of ligand–protein interactions throughout the simulation. In the 1J3K system (Figure 10A,B), both compounds exhibited dynamic hydrogen bonding behavior, with the number of interactions fluctuating between one and three over time. Compound 121 (Figure 10B) showed a slightly higher frequency of hydrogen bond formation compared to compound 11, indicating a more consistent interaction pattern within the binding site. For the 3IHZ system (Figure 10C,D), a distinct difference was observed between the two ligands. Compound 82 (Figure 10C) maintained a relatively stable number of hydrogen bonds, typically around two to three interactions, suggesting sustained contact with key residues. In contrast, compound 123 (Figure 10D) displayed greater variability, with occasional drops to lower interaction counts, reflecting a more dynamic binding behavior. These observations suggest that while all ligands are capable of forming stable hydrogen bond interactions, compound 121 in the 1J3K system and compound 82 in the 3IHZ system demonstrated more persistent interaction patterns. Such stability in hydrogen bonding may contribute significantly to maintaining ligand positioning within the active site during the simulation. Although compound 82 exhibited more persistent hydrogen bonding, this did not fully compensate for its higher structural fluctuations observed in the RMSD and SASA analyses, indicating that hydrogen bond count alone is not sufficient to ensure overall complex stability.

It is important to note that the stability of a protein–ligand complex cannot be assessed based on a single parameter. Although compound 82 exhibited persistent hydrogen bond interactions, its higher fluctuations in the RMSD and SASA analyses indicate a less stable dynamic behavior compared to compound 123. This highlights the importance of combining multiple descriptors to obtain a reliable evaluation of ligand stability. While molecular dynamics simulations provide detailed insights into structural stability and interaction behavior, the estimation of binding free energy is essential for quantifying ligand affinity. Therefore, MM/GBSA calculations were performed to further evaluate the energetic stability of the selected complexes.

#### 3.4.7. Integrated Assessment of Ligand Performance

Although individual molecular dynamics descriptors may favor different ligands, a comprehensive interpretation requires integrating all computational parameters rather than relying on a single metric. Table 5 summarizes the overall performance of the investigated compounds by combining docking affinity, dynamic stability, and interaction persistence.

Within the 1J3K system, compounds 11 and 121 both exhibited stable dynamic behavior and favorable docking characteristics. Compound 11 maintained a more consistent ligand orientation and slightly greater structural compactness, as reflected by ligand RMSD and Rg analyses. However, compound 121 demonstrated lower residue fluctuations and more persistent hydrogen-bond interactions, suggesting enhanced stabilization of the protein environment. Therefore, despite minor differences in compactness, compound 121 exhibited the most balanced overall profile for the 1J3K target.

For the 3IHZ system, compounds 82 and 123 displayed distinct dynamic characteristics. Compound 82 showed stronger hydrogen-bond persistence and lower residue fluctuations, indicating favorable interaction stability. Nevertheless, its elevated ligand RMSD and slight increase in Rg suggest greater conformational variability during the simulation. In contrast, compound 123 maintained a more stable ligand orientation and preserved compact structural behavior, supporting more consistent accommodation within the binding pocket. Consequently, compound 123 emerged as the most favorable overall candidate for the 3IHZ system.

These findings indicate that the global performance of a ligand should be interpreted through the combined contribution of docking, pharmacokinetic properties, and molecular dynamics descriptors, rather than by considering isolated parameters independently.

### 3.5. MM/GBSA Analysis

The MM/GBSA binding free energy results (Table 6) provide quantitative confirmation of the interaction strength observed in docking and molecular dynamics analyses. For the 1J3K system, compound 121 exhibited the most favorable binding energy (−77.56 kcal/mol), followed by compound 11 (−69.45 kcal/mol), indicating stronger interaction and enhanced stability of the 1J3K–121 complex. In the 3IHZ system, compound 123 showed a more favorable ΔG_binding (−55.78 kcal/mol) compared to compound 82 (−44.89 kcal/mol), confirming its superior stability. These findings are consistent with the RMSD and SASA results, which demonstrated improved dynamic behavior for compound 123. Overall, the agreement between MM/GBSA, molecular dynamics, and docking results strengthens the reliability of the predicted binding modes and highlights compounds 121 and 123 as the most promising candidates for further investigation.

### 3.6. Structural Characterization of the Most Promising Compounds

**Figure 11:**
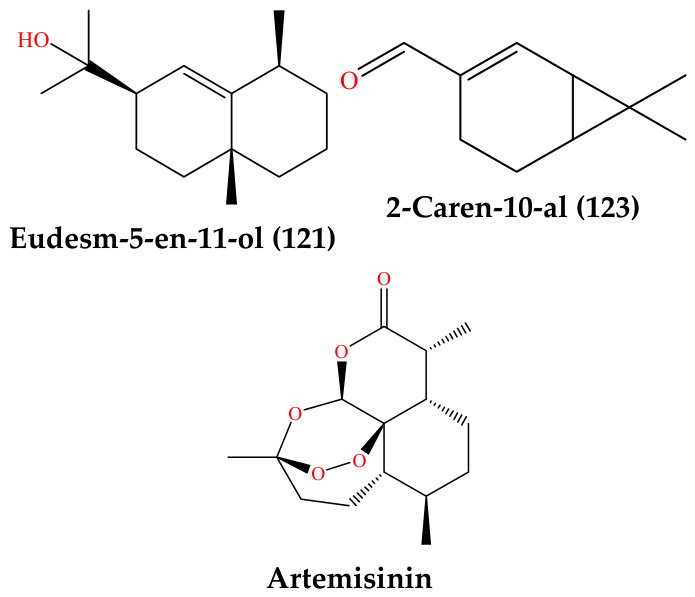
Two-dimensional chemical structures of the most promising compounds identified through the integrated computational analyses, namely Eudesm-5-en-11-ol (121), 2-Caren-10-al (123), and the reference antimalarial drug artemisinin.

To provide additional insight into the molecular basis underlying the computational performance of the most promising ligands, the two-dimensional chemical structures of the selected compounds and the reference drug are presented in Figure 11. Structural characterization may help identify key molecular features potentially associated with favorable binding affinity, interaction stability, and pharmacokinetic behavior observed during the docking, ADMET, and molecular dynamics analyses.

### 3.7. Structure–Interaction Relationship Analysis of the Most Promising Compounds

The structural analysis of the most promising compounds revealed molecular features potentially contributing to their favorable interaction profiles with the investigated targets. Eudesm-5-en-11-ol (121) possesses a hydrophobic sesquiterpene framework combined with a hydroxyl functional group, which may facilitate favorable accommodation within the binding cavity through hydrophobic contacts and hydrogen-bond interactions. This structural arrangement may contribute to its favorable binding affinity and stable dynamic behavior observed against the 1J3K target. In contrast, 2-Caren-10-al (123) contains a compact bicyclic scaffold and an aldehyde functional group that may support efficient orientation and interaction persistence within the 3IHZ binding site. Its relatively rigid framework may further contribute to maintaining stable protein–ligand interactions throughout the simulation period. Although artemisinin demonstrated comparatively limited hydrogen-bond interactions, its pharmacological activity is primarily attributed to the activation of its endoperoxide bridge, leading to reactive radical formation and oxidative damage within malaria parasites. Overall, the observed interaction profiles suggest that hydrophobic frameworks combined with suitable polar functional groups may represent favorable structural features contributing to the binding behavior and computational performance of the investigated compounds.

Despite these promising findings, several limitations should be acknowledged. Molecular docking provides only a qualitative estimation of binding affinity, and small differences in docking scores should be interpreted with caution. Molecular dynamics simulations supported the stability of ligand–protein complexes; however, the limited simulation time and lack of replicate runs may affect the robustness of the results. In addition, ADMET predictions are based on computational models and may not fully reflect in vivo pharmacokinetic behavior or toxicity. Furthermore, potential synergistic effects between phytoconstituents were not evaluated, although they may significantly contribute to biological activity in natural extracts. Therefore, experimental validation remains essential to confirm the predicted antileishmanial activity and pharmacological properties of the identified compounds.

## 4. Conclusions

This study provides a comprehensive in silico investigation of selected compounds targeting key proteins through an integrated approach combining molecular docking, pharmacokinetic prediction, and molecular dynamics simulations. Among the investigated compounds, Eudesm-5-en-11-ol (compound 121) and 2-Caren-10-al (compound 123) emerged as the most promising candidates, exhibiting a favorable balance between binding affinity, pharmacokinetic properties, and dynamic stability. In particular, Eudesm-5-en-11-ol demonstrated the most favorable binding behavior within the 1J3K system, while 2-Caren-10-al showed enhanced structural stability and consistent interaction patterns in the 3IHZ system. These findings highlight the potential of these naturally derived compounds as lead candidates for the further development of novel antimalarial agents. However, it is important to note that these findings are based on computational predictions and require further validation through in vitro and in vivo studies. Such studies are essential to confirm the biological activity and therapeutic potential of the identified compounds. They will also support their potential development as alternative treatments against *P. falciparum* and *P. vivax*.

## Figures and Tables

**Table 1:** Binding interactions of the selected molecules with the active-site residues of the 1J3K protein.

Molecule	Flexible Ligand Glide Gscore (kcal/mol)	Residues	Interaction	Rigid Ligand Glide Gscore (kcal/mol)	Residues	Interaction
11	−6.89	LEU164	Hydrogen bond	−6.25	TYR170	Hydrogen bond
		PHE58, ALA16, MET55, LEU46	π–Alkyl		PHE58, ALA16, MET55, LEU46	π–Alkyl
9	−6.18	LEU164, TYR170	Hydrogen bond	−6.16	LEU164, TYR170	Hydrogen bond
		ILE112, LEU46, PHE58, ALA16	π–Alkyl		ILE14, LEU46, LEU40, PHE58, ALA16	π–Alkyl
121	−6.53	LEU164	Hydrogen bond	−5.78	–	–
		ILE112, LEU46, PHE58, LEU40, MET55	π–Alkyl		ILE112, PRO113, LEU46, PHE58, LEU164	π–Alkyl
118	−4.35	TYR170	Hydrogen bond	−4.64	ARG122, ARG59	Hydrogen bond
		LEU119, PHE116, MET55, ILE112, PHE58	π–Alkyl		PRO113, LEU119, LEU164, PHE116, MET55, ILE112, PHE58	π–Alkyl
		CYS15	Carbon hydrogen bond		–	–
Artemisinin	−5.52	TYR170	Hydrogen bond	−6.12	TYR170, ASN108	Hydrogen bond
		LEU46, MET55, PHE58, ALA16	π–Alkyl		LEU46, LEU40, PHE58, ALA16, ILE112	π–Alkyl
		GLY166, LEU164, CYS15	Carbon hydrogen bond		–	–

**Table 2:** Binding interactions of the molecules with the active residues of the 3IHZ protein.

Molecule	Flexible Ligand Glide Gscore (kcal/mol)	Residues	Interaction	Rigid Ligand Glide Gscore (kcal/mol)	Residues	Interaction
123	−6.81	ILE74	Hydrogen bond	−6.98	ILE74	Hydrogen bond
		VAL73	Carbon hydrogen bond		VAL73	Carbon hydrogen bond
		PHE117, PHE54, PHE64, TYR43, TRP77	π–Alkyl		PHE117, PHE54, TYR43, TYR100, TRP77	π–Alkyl
132	−6.80	ILE74	Hydrogen bond	−6.82	ILE74	Hydrogen bond
		PHE117, TRP77	π–Alkyl		PHE117, TRP77	π–Alkyl
		PHE64, TYR43	π–Sigma		PHE64, TYR43	π–Sigma
H1	−5.91	ILE74	Hydrogen bond	−6.22	ILE74	Hydrogen bond
		ILE109, CYS106, TYR100, TYR43, PHE117	π–Alkyl		ILE109, CYS106, TYR100, TYR43, PHE117	π–Alkyl
82	−5.50	ASP55	Hydrogen bond	−5.76	ASP55	Hydrogen bond
		TYR43	Unfavorable donor–donor		TYR43	Unfavorable donor–donor
		ILE74, PHE64, VAL73, TRP77, ILE109, PHE117, PHE54, TYR100	π–Alkyl		ILE74, PHE64, VAL73, TRP77, ILE109, PHE117, PHE54, TYR100	π–Alkyl
Artemisinin	−4.95	PHE64, VAL73, TYR43	π–Alkyl	−4.92	PHE64, VAL73, TYR43	π–Alkyl
		TRP77	π–Sigma		TRP77	π–Sigma

**Table 3:** Predicted ADMET properties of selected ligands and reference drug against Plasmodium vivax FK506-binding protein 35 (3IHZ) using QikProp.

Compound	MW (g/mol)	QPlogP O/w	QPlogS	Caco-2 (nm/s)	QPlogBB	% Oral Absorption	Lipinski Violations	HERG (log IC50)
82	152.23	2.25	−2.27	3983	0.11	100	0	−3.12
132	154.20	1.16	−1.37	2287	0.06	94	0	−2.33
123	150.22	1.88	−2.28	2046	−0.07	100	0	−3.01
H1	402.52	4.93	−4.60	695	−1.76	100	0	−4.62
Artemisinin	282.33	1.75	−2.27	1959	−0.006	96	0	−2.63

**Table 4:** Predicted ADMET properties of selected ligands and reference drug against Plasmodium falciparum dihydrofolate reductase–thymidylate synthase (1J3K) using QikProp.

Compound	MW (g/mol)	QPlogPo/w	QPlogS	Caco-2 (nm/s)	QPlogBB	% Oral Absorption	Lipinski Violations	HERG (log IC50)
9	222.37	3.79	−4.00	4437	0.22	100	0	−2.58
121	222.37	3.99	−4.21	4671	0.16	100	0	−2.95
11	222.37	3.95	−4.15	4635	0.16	100	0	−2.90
118	222.37	4.02	−4.45	5585	0.29	100	0	−3.08
Artemisinin	282.33	1.75	−2.27	1959	−0.006	96	0	−2.63

**Table 5:** Integrated summary of computational performance and overall assessment of the investigated ligands.

Compound	Target	Docking Affinity	RMSD Behavior	RMSF Profile	Rg Profile	H-Bond Persistence	Overall Assessment
11	1J3K	Favorable	Stable ligand orientation	Moderate fluctuations	More compact structure	Moderate	Good stability profile
121	1J3K	Favorable	Stable protein profile with initial ligand adaptation	Lower fluctuations	Slightly less compact	Higher persistence	Most favorable overall for 1J3K
82	3IHZ	Favorable	High ligand fluctuations	Lower residue mobility	Slight late increase	High persistence	Stable interactions but dynamic variability
123	3IHZ	Favorable	Stable ligand orientation	Moderate localized flexibility	Stable compactness	Moderate	Most favorable overall for 3IHZ
Artemisinin	Reference	Lower affinity	Variable	Moderate	Stable	Limited	Reference profile

**Table 6:** Binding free energy of selected complexes.

Complex	ΔG_Binding (kcal/mol)
1J3K–11	−69.45
1J3K–121	−77.56
3IHZ–82	−44.89
3IHZ–123	−55.78

## Data Availability

The original contributions presented in this study are included in the article/Supplementary Materials. Further inquiries can be directed to the corresponding authors.

## Supplementary Materials

The following supporting information can be downloaded at: https://www.mdpi.com/article/10.3390/cimb48070701/s1.
