# Supplementary material for: Computational Screening of Djiboutian Medicinal Plants Reveals Potential Dual Inhibitors Against Plasmodium falciparum and Plasmodium vivax
Source: Curr Issues Mol Biol. 2026 Jul 10;48(7):701. doi: 10.3390/cimb48070701 (PMC13407601; doi:10.3390/cimb48070701)
Supplement: Supplementary file 1 [file cimb-48-00701-s001.zip › cimb-4401026-supplementary.pdf]

Supplementary Table S1. Chemical dataset of phytoconstituents from Djiboutian medicinal plants

Plant	Cocmpound	No	Smiles	Extract	Part of the Plant and Collection Zone
Tagetes minuta L.	D-Limonene	1	CC1=CC[C@@H](CC1)C(=C)C	stems, leaves and flowers	Day (North Djibouti) (11◦45.180 N; 42◦37.730
	Camphor	2	CC1(C2CCC1(C(=O)C2)C)C		
	Estragole	3	COC1=CC=C(C=C1)CC=C		
	Verbenone	4	CC1=CC(=O)[C@@H]2C[C@H]1C2(C)C		
	(Z)-Tagetenone	5	CC(=CC(=O)/C=C(/C)\\C=C)C		
	Piperitone	6	CC1=CC(=O)C(CC1)C(C)C		
	2-Undecanone	7	CCCCCCCCCC(=O)C		
	Benzene, 2-tert-butyl-1,4-dimethoxy-	8	CC(C)(C)C1=C(C=CC(=C1)OC)OC		
	(-)—Spathulenol	9	C[C@@]1(CC[C@@H]2[C@@H]1[C@H]3[C@H](C3(C)C)CCC2=C)O		
	Cubenol	10	C[C@@H]1CC[C@H]([C@H]2[C@]1(CCC(=C2)C)O)C(C)C		
	τ-Cadinol	11	CC1=C[C@H]2[C@@H](CC[C@]([C@@H]2CC1)(C)O)C(C)C		
	Dihydrotagetone	12	CC(C)CC(=O)CC(C)C=C		

	Artemisia	13	CC(=CC(=O)C(C)(C)C=C)C		
Lavandula coronopifolia L.	α-Pinene	14	CC1=CCC2CC1C2(C)C	stems, leaves and flowers	Day (North Djibouti) (11◦45.180 N; 42◦37.730
	Linalol	15	CC(=CCCC(C)(C=C)O)C		
	Isopinocarveol	16	CC1([C@@H]2C[C@H]1C(=C)[C@@H](C2)O)C		
	Verbenol	17	CC1=CC(C2CC1C2(C)C)O		
	Myrtenol	18	CC1(C2CC=C(C1C2)CO)C		
	D-Verbenone	19	CC1=CC(=O)[C@@H]2C[C@H]1C2(C)C		
	Bornyl Acetate	20	CC(=O)O[C@@H]1C[C@@H]2CC[C@]1(C2(C)C)C		
	α-Terpineol Acetate	21	CC1=CC(CCC1)C(C)(C)OC(=O)C		
	Copaene	22	CC1=CC[C@H]2[C@H]3[C@@H]1[C@@]2(CC[C@H]3C(C)C)C		
	Cubenene	23	C12C3C4C1=C5C2C3=C45		
	Zingiberene	24	CC1=CC[C@@H](C=C1)[C@@H](C)CCC=C(C)C		
	Cis-Caryophyllene	25	C/C/1=C/CCC(=C)[C@H]2CC([C@@H]2CC1)(C)C		
	α-Bergamotene	26	CC1=CCC2CC1C2(C)CCC=C(C)C		
	β-Sesquiphellandrene	27	C[C@@H](CCC=C(C)C)[C@H]1CCC(=C)C=C1		
	Humulene	28	C/C/1=C\CC(/C=C/C/C(=C/CC1)/C)(C)C		
	Aromadendrene	29	C[C@@H]1CC[C@@H]2[C@@H]1[C@H]3[C@H](C3(C)C)CCC2=C		

	Germacrene D	30	C/C/1=C\\CCC(=C)/C=C/[C@@H](CC1)C(C)C		
	β-Eudesmene	31	CC(=C)[C@@H]1CC[C@]2(CCCC(=C)[C@@H]2C1)C		
	Methyl (2E)-2-nonenoate	32	CCCCCC/C=C/C(=O)OC		
	Bisabolene	33	CC1=CCC(=C(C)CCC=C(C)C)CC1		
	τ-Cadinene	34	CC1=C[C@H]2[C@H](CC1)C(=C)CC[C@@H]2C(C)C		
	δ-Cadinene	35	CC1=C[C@H]2[C@@H](CCC(=C2CC1)C)C(C)C		
	α-Panasinsen	36	CC1=CCCC2(C13CC(C3CC2)(C)C)C		
	α-Caryophyllene	37	C/C/1=C\CC(/C=C/C/C(=C/CC1)/C)(C)C		
	β- Elemol	38	CC([C@@H]1C[C@H](C(C)(O)C)CC[C@]1(C=C)C)=C		
	Epiglobulol	39	C[C@@H]1CC[C@@H]2[C@@H]1[C@H]3[C@@H](CC[C@@]2(O)C)C3(C)C		
	Dehydronerolidol	40	CC(C)=CCC/C(C)=C/CC[C@@](C#C)(O)C		
	Farnesyl acetone	41	CC(=CCC/C(=C/CC/C(=C/CCC(=O)C)/C)/C)C		
	Caryophyllene Oxide	42	C[C@@]12CC[C@@H]3[C@H](CC3(C)C)C(=C)CC[C@H]1O2		
	12-Heptadecyn-1-ol	43	CCCCC#CCCCCCCCCCCO		
	Longipinane	44	CC1CCC2C3C1C2(CCCC3(C)C)C		
	Humulene Epoxide	45	C/C/1=C\CC(/C=C/C[C@@]2([C@H](O2)CC1)C)(C)C		
	α-Cadinol	46	CC1=C[C@H]2[C@@H](CC[C@@]([C@@H]2CC1)(C)O)C(C)C		

	10-epi-β-eudesmol	47	C[C@H]1C[C@@H]([C@H]([C@]2([C@H]1C[C@@H](CC2)C(=C)C)C)O)O		
	Viridiflorol	48	C[C@@H]1CC[C@H]2[C@@H]1[C@H]3[C@H](C3(C)C)CC[C@]2(C)O		
	α-Bisabolol	49	CC1=CC[C@H](CC1)[C@](C)(CCC=C(C)C)O		
	Isolongifolanone	50	CC1(CCC(=O)C2[C@@]13CC[C@@H](C3)C2(C)C)C		
	Trans-Farnesol	51	CC(=CCC/C(=C/CC/C(=C/CO)/C)/C)C		
R. chalepensis L.	2-nonanone	52	CCCCCCCC(=O)C	Aerial parts	Foret de Day National Park of the Republic of Djibouti (11◦45003.600 N 42◦41013.200 E)
	Cyclohexene, 3,4-diethenyl-3-methyl-	53	CC1(C=CCCC1C=C)C=C		
	2-decanone	54	CCCCCCCCC(=O)C		
	Octyl acetate	55	CCCCCCCCOC(=O)C		
	Chloromethyl octyl ether	56	CCCCCCCCOCCl		
	2-undecanol	57	CCCCCCCCCC(C)O		
	Stearic acid	58	CCCCCCCCCCCCCCCCCC(=O)O		
	2-acetoxytridecane	59	CCCCCCCCCCCC(C)OC(=O)C		
	2-dodecanone	60	CCCCCCCCCCC(=O)C		
	1-Methyl-2-decalone	61	CC1C2CCCCC2CCC1=O		
	2-acetoxytetradecane	62	CCCCCCCCCCCCC(C)OC(=O)C		

	Bicyclosesquiphellandrene	63	C[C@@H]1CC[C@H](C2=CC(=C)CC[C@H]12)C(C)C		
	5-ketobornyl acetate	64	CC(=O)OC1CC2C(=O)CC1(C2(C)C)C		
	Germacrene D	65	C/C/1=C\\CCC(=C)/C=C/[C@@H](CC1)C(C)C		
	2-tridecanone	66	CCCCCCCCCCCC(=O)C		
	B- elemol	67	CC(=C)[C@@H]1C[C@@H](CC[C@@]1(C)C=C)C(C)(C)O		
	Nerolidol	68	CC(=CCC/C(=C/CCC(C)(C=C)O)/C)C		
	T-cadinol	69	CC1=C[C@H]2[C@@H](CC[C@]([C@@H]2CC1)(C)O)C(C)C		
	3-Heptenoic acid, 7-phenyl-, ethyl ester,	70	CCOC(=O)C/C=C/CCCC1=CC=CC=C1		
	Dihydrosafrol	71	CCCC1=CC2=C(C=C1)OCO2		
	Piperonyl acetone	72	CC(=O)/C=C/C1=CC2=C(C=C1)OCO2		
	3-Methyl-2-butenoic acid, cyclobutyl ester	73	CC(=CC(=O)OC1CCC1)C		
Ocimum basilicum L.	Linalol	15	CC(=CCCC(C)(C=C)O)C	Aerial parts	Ambouli (11°33′29.3″ N 43°08′47.7″ E)
	Estragole	3	COC1=CC=C(C=C1)CC=C		
	α-bergamotene	26	CC1=CCC2CC1C2(C)CCC=C(C)C		
	τ-cadinol	11	CC1=C[C@H]2[C@@H](CC[C@]([C@@H]2CC1)(C)O)C(C)C		
	δ-cadinene	35	CC1=C[C@H]2[C@@H](CCC(=C2CC1)C)C(C)C		

	Cis-caryophyllene	25	C/C/1=C/CCC(=C)[C@H]2CC([C@@H]2CC1)(C)C		
	Bornyl acetate	20	CC(=O)O[C@@H]1C[C@@H]2CC[C@]1(C2(C)C)C		
	Elemene	74	CC(C)C1=C[C@@H]([C@@](CC1)(C)C=C)C(=C)C		
	Camphor	2	CC1(C2CCC1(C(=O)C2)C)C		
	Epiglobulol	39	C[C@@H]1CC[C@@H]2[C@@H]1[C@H]3[C@@H](CC[C@@]2(O)C)C3(C)C		
	Viridiflorol	48	C[C@@H]1CC[C@H]2[C@@H]1[C@H]3[C@H](C3(C)C)CC[C@]2(C)O		
	10-epi-β-eudesmol	47	C[C@H]1C[C@@H]([C@H]([C@]2([C@H]1C[C@@H](CC2)C(=C)C)C)O)O		
	Caryophyllene oxide	42	C[C@@]12CC[C@@H]3[C@H](CC3(C)C)C(=C)CC[C@H]1O2		
	Humulene	28	C/C/1=C\CC(/C=C/C/C(=C/CC1)/C)(C)C		
	Cadinol	75	CC1=CC2[C@@H](CC[C@@](C2CC1)(C)O)C(C)C		
	(-)-Spathulenol	76	C[C@]1(CC[C@H]2[C@H]1[C@@H]3[C@@H](C3(C)C)CCC2=C)O		
	Linalol oxide	77	CC1(C(O1)CCC(C)(C=C)O)C		
	α-bulnesene	78	C[C@H]1CCC2=C(CC[C@H](C[C@@H]12)C(=C)C)C		
	Epoxide farnesene	79	CC(=CCC/C(=C/CCC(=C)C1CO1)/C)C		
	β-elemol	38	CC([C@@H]1C[C@H](C(C)(O)C)CC[C@]1(C=C)C)=C		
	Jasmone	80	CC1=C(C/C=C\CC)C(CC1)=O		
	β-sesquiphellandrene	27	C[C@@H](CCC=C(C)C)[C@H]1CCC(=C)C=C1		

	Nerolidol	68	CC(=CCC/C(=C/CCC(C)(C=C)O)/C)C		
	Humulene epoxide	45	C/C/1=C\CC(/C=C/C[C@@]2([C@H](O2)CC1)C)(C)C		
	Octyl acetate	55	CCCCCCCCOC(=O)C		
	Bisabolene	33	CC1=CCC(=C(C)CCC=C(C)C)CC1		
	α-farnesene	81	C=C/C(C)=C/C/C=C(CC/C=C(C)/C)\C		
	Cis-carveol	82	C=C(C)[C@@H]1C[C@H](O)C(C)=CC1		
	Cyclopentanone, 3-[3,5-decadienyl]-,	83	O=C1CC(CC/C=C/C=C/CCCC)CC1		
	Isolongifolanone	50	CC1(CCC(=O)C2[C@@]13CC[C@@H](C3)C2(C)C)C		
	Cis-3-hexenyl isobutyrate	84	CC(C)C(OCC/C=C\CC)=O		
	Copaene	22	CC1=CC[C@H]2[C@H]3[C@@H]1[C@@]2(CC[C@H]3C(C)C)C		
	Methyl isoeugenol	85	COC1=CC(C=CC)=CC=C1OC		
	Bergamotene trans	86	CC(=CCC[C@@]1([C@H]2CCC(=C)[C@@H]1C2)C)C		
	β-eudesmene	31	CC(=C)[C@@H]1CC[C@]2(CCCC(=C)[C@@H]2C1)C		
	Spiro [4.5]decane, 6-methylene-	87	C=C(CCCC1)C21CCCC2		
	Carvotanacetol	88	CC1=CC[C@@H](C[C@@H]1O)C(C)C	Aerial parts	Day (11°45′07.1″
	Estragole	3	COC1=CC=C(C=C1)CC=C		

Ocimum americanum L.	α-bergamotene	26	CC1=CCC2CC1C2(C)CCC=C(C)C		N 42°41′50.1″ E)
	τ-cadinol	11	CC1=C[C@H]2[C@@H](CC[C@]([C@@H]2CC1)(C)O)C(C)C		
	δ-cadinene	35	CC1=C[C@H]2[C@@H](CCC(=C2CC1)C)C(C)C		
	Elemene	74	CC(C)C1=C[C@@H]([C@@](CC1)(C)C=C)C(=C)C		
	Germacrene D	30	C/C/1=C\\CCC(=C)/C=C/[C@@H](CC1)C(C)C		
	β-elemol	38	CC([C@@H]1C[C@H](C(C)(O)C)CC[C@]1(C=C)C)=C		
	10-epi-β-eudesmol	47	C[C@H]1C[C@@H]([C@H]([C@]2([C@H]1C[C@@H](CC2)C(=C)C)C)O)O		
	Bornyl acetate	20	CC(=O)O[C@@H]1C[C@@H]2CC[C@]1(C2(C)C)C		
	Camphor	2	CC1(C2CCC1(C(=O)C2)C)C		
	Cadinol	75	CC1=CC2[C@@H](CC[C@@](C2CC1)(C)O)C(C)C		
	Trans-4-methoxycinnamaldehyde	89	O=C/C=C/C1=CC=C(OC)C=C1		
	α-bulnesene	78	C[C@H]1CCC2=C(CC[C@H](C[C@@H]12)C(=C)C)C		
	β-cedrene	90	C[C@@H]1CC[C@@H]2[C@]13CCC(=C)[C@H](C3)C2(C)C		
	Humulene	28	C/C/1=C\CC(/C=C/C/C(=C/CC1)/C)(C)C		
	Linalol oxide	77	CC1(C(O1)CCC(C)(C=C)O)C		
	Jasmone	80	CC1=C(C/C=C\CC)C(CC1)=O		

	Nerolidol	68	CC(=CCC/C(=C/CCC(C)(C=C)O)/C)C		
	3,7-octadiene-2,6-diol, 2,6-dimethyl-	91	CC(O)(C)C=CCC(O)(C)C=C		
	Piperitone	6	CC1=CC(=O)C(CC1)C(C)C		
	Caryophyllene oxide	42	C[C@@]12CC[C@@H]3[C@H](CC3(C)C)C(=C)CC[C@H]1O2		
	Octyl acetate	55	CCCCCCCCOC(=O)C		
	β-eudesmene	31	CC(=C)[C@@H]1CC[C@]2(CCCC(=C)[C@@H]2C1)C		
	Oxirane, 2-(hexyn-1-yl)-3-methoxymethylene-	92	COC=C1C(C#CCCCC)O1		
	1,5,9-trimethyl cyclododecatriene	93	CC1CC/C=C(C)\C=C\C=C(C)/CCC1		
	Beta-bourbonene	94	CC(C)[C@@H]1CC[C@@]2([C@H]1[C@@H]3[C@H]2CCC3=C)C		
	Bicyclosesquiphellandrene	63	C[C@@H]1CC[C@H](C2=CC(=C)CC[C@H]12)C(C)C		
	α-caryophyllene	37	C/C/1=C\CC(/C=C/C/C(=C/CC1)/C)(C)C		
	Methyl crotonate	95	C/C=C/C(OC)=O		
	Cis-caryophyllene	25	C/C/1=C/CCC(=C)[C@H]2CC([C@@H]2CC1)(C)C		
	Bisabolene	33	CC1=CCC(=C(C)CCC=C(C)C)CC1		
	Isolongifolanone	50	CC1(CCC(=O)C2[C@@]13CC[C@@H](C3)C2(C)C)C		

	Copaene	22	CC1=CC[C@H]2[C@H]3[C@@H]1[C@@]2(CC[C@H]3C(C)C)C		
	Methyl isoeugenol	85	COC1=CC(C=CC)=CC=C1OC		
	Octen-1-ol, acetate	96	CCCCCCC=COC(C)=O		
	Bergamol	97	C/C(C)=C\CCC(C)(C=C)OC(C)=O		
	γ-eudesmol	98	CC1=C2C[C@@H](CC[C@]2(CCC1)C)C(C)(C)O		
	Eudesma-4,11-dien-2-ol	99	CC(CC(O)C[C@@](C)1CC2)=C1C[C@@H]2C(C)=C		
	Longipinocarveol	100	CC1(CCCC2(C3C1C2C(=C)C(C3)O)C)C		
	(-)-Spathulenol	76	C[C@]1(CC[C@H]2[C@H]1[C@@H]3[C@@H](C3(C)C)CCC2=C)O		
	β-sesquiphellandrene	27	C[C@@H](CCC=C(C)C)[C@H]1CCC(=C)C=C1		
	Cyclopentanone, 3-[3,5-decadienyl]-,	83	O=C1CC(CC/C=C/C=C/CCCC)CC1		
	Bergamotene trans	86	CC(=CCC[C@@]1([C@H]2CCC(=C)[C@@H]1C2)C)C		
Cymbopogon schoenanthus (L.) Spreng.	3-Isopropenyl-5-methyl-1-cyclohexene	101	CC1CC(C(C)=C)C=CC1	Plant materials	Mouloud region (south-west of Djibouti; 11°10′18.3″ N 42°30′01.4″ E)
	D-Limonene	1	CC1=CC[C@@H](CC1)C(=C)C		
	α-Terpineol	102	CC(O)(C1CCC(C)=CC1)C		
	β- elemol	38	CC([C@@H]1C[C@H](C(C)(O)C)CC[C@]1(C=C)C)=C		
	2-p-Menthen-1-ol	103	CC1(O)CCC(C(C)C)C=C1		

	Geraniol acetate	104	C/C(CC/C=C(C)\C)=C\COC(C)=O		
	Terpineol, cis-,beta,-	105	CC(=C)C1CCC(CC1)(C)O		
	10-epi-β-eudesmol	47	C[C@H]1C[C@@H]([C@H]([C@]2([C@H]1C[C@@H](CC2)C(=C)C)C)O)O		
	4-Nonanone	106	CCCC(CCCCC)=O		
	cis-Piperitol	107	CC1=C[C@@H]([C@@H](CC1)C(C)C)O		
	β-trans-Ocimene	108	C=C/C(C)=C/C/C=C(C)/C		
	trans-Piperitol	109	CC1=C[C@@H]([C@H](CC1)C(C)C)O		
	Eudesm-7(11)-en-4-ol	110	C[C@]([C@](C/1)2[H])(O)CCC[C@]2(C)CCC1=C(C)\C		
	Caryophyllene oxide	42	C[C@@]12CC[C@@H]3[C@H](CC3(C)C)C(=C)CC[C@H]1O2		
	Ocimene	111	C=CC(C)=CC/C=C(C)/C		
	Cyclohexene, 3-acetoxy-4-(1-hydroxy-1-methylethyl)-1-methyl-	112	CC1=CC(OC(C)=O)C(C(C)(O)C)CC1		
	Elemene	74	CC(C)C1=C[C@@H]([C@@](CC1)(C)C=C)C(=C)C		
	Cis-caryophyllene	25	C/C/1=C/CCC(=C)[C@H]2CC([C@@H]2CC1)(C)C		
	Anethofuran	113	CC(CC1)=C[C@@]2([H])[C@]1([H])[C@H](C)CO2		
	α-Phellandren-8-ol	114	CC1=CCC(C=C1)C(C)(C)O		
	γ-eudesmol	98	CC1=C2C[C@@H](CC[C@]2(CCC1)C)C(C)(C)O		

	Viridiflorol	48	C[C@@H]1CC[C@H]2[C@@H]1[C@H]3[C@H](C3(C)C)CC[C@]2(C)O		
	Isogeraniol	115	C/C(C)=C/C/C=C(C)\CCO		
	δ-cadinene	35	CC1=C[C@H]2[C@@H](CCC(=C2CC1)C)C(C)C		
	τ-cadinol	11	CC1=C[C@H]2[C@@H](CC[C@]([C@@H]2CC1)(C)O)C(C)C		
	β-Phellandrene	116	CC(C)C1CCC(=C)C=C1		
	L-Fenchone	117	C[C@@]12CC[C@@H](C1)C(C2=O)(C)C		
	Neryl acetate	118	CC(OC/C=C(CC/C=C(C)/C)/C)=O		
	cis-Geraniol	119	C/C(CC/C=C(C)/C)=C/CO		
	2-Acetoxy-1,8-cineole	120	CC(OC1CC2CCC1(C)OC2(C)C)=O		
	Eudesm-5-en-11-ol	121	C[C@H]1CCC[C@]2(C1=C[C@@H](CC2)C(C)(C)O)C		
	4-Undecanone	122	CCCCCCCC(=O)CCC		
	2-Caren-10-al	123	CC1(C2C1C=C(CC2)C=O)C		
	1-Tetradecyne	124	C#CCCCCCCCCCCCC		
	4-Dodecanone, 11-methyl-	125	CCCC(CCCCCCC(C)C)=O		
	Germacrene D	30	C/C/1=C\\CCC(=C)/C=C/[C@@H](CC1)C(C)C		
	τ-cadinene	34	CC1=C[C@H]2[C@H](CC1)C(=C)CC[C@@H]2C(C)C		
	Eudesma-4,11-dien-2-ol	99	CC(CC(O)C[C@@](C)1CC2)=C1C[C@@H]2C(C)=C		

	longipinocarveol	100	CC1(CCCC2(C3C1C2C(=C)C(C3)O)C)C		
	α-Phellandrene	126	CC1=CCC(C=C1)C(C)C		
	cis-p-menth-2-en-1-ol	127	CC(C)[C@H]1CC[C@](C=C1)(C)O		
	Cyclohexene, 2-ethenyl-1,3,3-trimethyl-	128	CC1=C(C(CCC1)(C)C)C=C		
	α-Bergamotene	26	CC1=CCC2CC1C2(C)CCC=C(C)C		
	β-eudesmene	31	CC(=C)[C@@H]1CC[C@]2(CCCC(=C)[C@@H]2C1)C		
	Methyl (2E)-2-nonenoate	32	CCCCCC/C=C/C(=O)OC		
	Cis-carveol	82	C=C(C)[C@@H]1C[C@H](O)C(C)=CC1		
	Trans-2-Hydroxy-1,8-cineole	129	CC1(C2CCC(O1)(C(C2)O)C)C		
Nepeta azurea R.Br. ex Benth	Methyl (2E)-2-nonenoate	32	CCCCCC/C=C/C(=O)OC	Plant materials	Day region (north of Djibouti; 11°44′46.6″ N 42°41′03.5″ E)
	Phellandral	130	CC(C)C1CCC(=CC1)C=O		
	1-Methyl-2-decalone	61	CC1C2CCCCC2CCC1=O		
	Cis-caryophyllene	25	C/C/1=C/CCC(=C)[C@H]2CC([C@@H]2CC1)(C)C		
	Cyclohexanol, acetate	131	CC(=O)OC1CCCCC1		
	Boschnialactone	132	C[C@@H]1CC[C@H]2[C@@H]1COC(=O)C2		
	Caryophyllene oxide	42	C[C@@]12CC[C@@H]3[C@H](CC3(C)C)C(=C)CC[C@H]1O2		

	L-calamenene	133	C[C@H]1CC[C@H](C2=C1C=CC(=C2)C)C(C)C		
	Oplopanone	134	CC(C)[C@@H]1CC[C@@]([C@H]2[C@H]1[C@H](CC2)C(=O)C)(C)O		
	piperitone	6	CC1=CC(=O)C(CC1)C(C)C		
	Isogeraniol	115	C/C(C)=C/C/C=C(C)\CCO		
	Cubenol	10	C[C@@H]1CC[C@H]([C@H]2[C@]1(CCC(=C2)C)O)C(C)C		
	Viridiflorol	48	C[C@@H]1CC[C@H]2[C@@H]1[C@H]3[C@H](C3(C)C)CC[C@]2(C)O		
	Limonene oxide	135	CC(=C)C1CCC2(C(C1)O2)C		
	2-Undecanone	7	CCCCCCCCC(=O)C		
	1-Tetradecyne	124	C#CCCCCCCCCCCCC		
	β-Bourbonene	136	CC(C)[C@@H]1CC[C@@]2([C@H]1[C@@H]3[C@H]2CCC3=C)C		
	Elemene	74	CC(C)C1=C[C@@H]([C@@](CC1)(C)C=C)C(=C)C		
	Linalol	15	CC(=CCCC(C)(C=C)O)C		

ID	SMILES
A1	OC(CCCCCCCCCCCCCCCCCCCCC)=O
A2	CSi(C)OSi(C)C
A3	OC(CCCCCCCCCCCCCCCCCCC)=O
A4	O=C(CCCCCCC/C=C\CCCCCCCC)O
A5	O=C/C(CCCCCC)=C\C1=CC=CC=C1
A6	O=C1CCCCCC1

A7	OC@@HC@HCC
A8	C/C(CC/C=C(C)/CCC=C(C)C)=C\CC/C=C(C)/CC/C=C(C)/CCC=C(C)C
A9	BrC@HCCCCCCCCCCCC
A10	ClC@@(C(Cl)=C2Cl)C@H[C@H]3[C@@]12Cl
A11	ClC@(C(Cl)=C2Cl)C@@H[C@@H]4[C@]12Cl
B1	CSi(C)OSi(C)C
B2	FC1=CC=C(C=C1)C2=CC=CC=C2
B3	ClC(C(OCC(OC)=O)=C1)=CC(Cl)=C1Cl
B4	C(C1=CC=CC=C1)(C=C2)=CC=C2OC3=CC=CC=C3
B5	ClC(C=C1)=C(C=C1Cl)N+=O
B6	CSi(C)C(C=C1Si(C)C)=C(C=C1)Si(C)C
B7	OC1=CC=C(C=C1)SC(C=C2)=CC=C2O
B8	OC(CCCCCCC/C=C\CCCCCCCC)=O
B9	CSi(C)C
B10	O=C(CCCCCCCCCCCCCCC)OCCCCCCCC
B11	ClC1=CC(C(C2=CC=CC=C2)=NC3)=C(C=C1)N(C)C3=O
C1	O=C(CCCCCCC/C=C\CCCCCCCC)OCCCC
C2	OC@@(C)CCCC(C)C
C4	O=C/C(CCCCCC)=C/C1=CC=CC=C1
C5	OC@HCCCCCCCCCCC
C6	ClC@@(C(Cl)=C2Cl)[C@H]3C@@H[C@@H]4[C@H]5O[C@H]5[C@H]3C4
C7	ClC@@(C(Cl)=C2Cl)[C@H]3C@@H[C@H]4[C@@H]5O[C@@H]5[C@@H]3C4
C8	ClC@@(C(Cl)=C2Cl)C@@HC@@H[C@@]12Cl
C9	O=C(CCCCCCCCC=C)OCCCC
C10	ClC1=C(C=CC(Cl)=C1)OC(C=C2)=CC(C(OC)=O)=C2N+=O
D1	C[Si]1(C)OSi(C)OSi(C)OSi(C)O1
D2	O=C(CCCCC)OC1CCCCC1
D3	OCCC#CCCCCC
D5	C=CCCCCCCCCCCC=C

D6	[O-]N+=O
D8	O=C(CC)CCCCCCCCCCCCC
D9	O=C(CCCCCCCCCCCCCCCCC)OCCCC
D10	CC@HNC
E1	ClC1=CC=CC(N+=O)=C1
E2	OCCOCCOCC
E3	ClC(C=C(C=C1)NC(OC)=O)=C1Cl
E4	OC(CCCCCCCCC#C)=O
E5	C=CCOC1=NC(OCC=C)=NC(OCC=C)=N1
E6	ClC1=CC=C(C=O)S1
E7	C/C(CC=C)=C\CC
E8	ClC1=C(C=CC(Cl)=C1)OP@@(OCC)=S
E9	BrC1CCCC1
F1	CC(C)=CCCC=C
F2	OC(CCCCCCCCC)=O
F3	O=C(C)/C=C/C1=C(C)CCCC1(C)C
F5	ClC(Cl)(C[C@@]1(C2=CC(Cl)=CC(Cl)=C2)OC1)Cl
F6	CCSn(CC)CC
F7	ClCCCCCCCCCCCCCCCCCCCCC
G1	S=C=NC
G2	VC/C=C/C(C=C1)=CC=C1OC
G3	ClC1=C(C(Cl)=O)C=CC(Cl)=C1
G4	ClC@(C(Cl)=C2Cl)C@@H[C@@H]3[C@]12Cl
G6	OC@HC(C)(C)C
G7	OC/C=C(C)/CC/C=C(C)/CCC=C(C)C
H1	OC(CC(OCCCCC)=O)(C(OCCCCC)=O)CC(OCCCCC)=O
H2	OC(CCC1CCCCC1)=O
H3	OC1(CN+=O)CCCCC1
H4	C1(C2=CSN=C2)=CC=CC=C1
H5	BrC(C(C(C)C)=CC(C(C)C)=C1)=C1C(C)C
H6	CCCCCCCCCCCCCCCC=C

I1	ClC1=CC(Cl)=CC=C1
I2	OC(CCCC)=O
I3	OC@HCCCCCCCCC
I4	[O-]N+=O
I5	CCCCCCCCCCCCCCCCCCCCCCCCCCCC
I6	OCC1CCCCCCC1
I7	OC@HCCC
J1	O=C(C)CCCCCCCCCCC
J2	OC@@(C=C)CC/C=C(C)/CCC=C(C)C
J3	C[C@]1(C[C@@H]2C3)C[C@]3(C)CC@@HC1
J4	CC1=C2N3C(SC2=CC=C1)=NN=C3
J5	O=C1C(C(C)C)=CC(C(C)=C1)=O
J6	BrC1([C@H]2[C@H]1CCCCCC2)Br
J7	C12=CC=CC=C1NCCC2
J8	C#CCCCCCCC#C
J9	O=C(CCCCCCCCCCCCCCC(OC)=O)OC
J10	OC(C1CCCC1)=O
J11	BrCCCCCCCCCCC(O)=O
